# Evaluation of an ultrasensitive HRP2–based rapid diagnostic test for detection of asymptomatic *Plasmodium falciparum* parasitaemia among children in western Kenya

**DOI:** 10.1186/s12936-022-04351-y

**Published:** 2022-11-16

**Authors:** Lindsey B. Turnbull, George Ayodo, Veronicah Knight, Chandy C. John, Megan S. McHenry, Tuan M. Tran

**Affiliations:** 1 Ryan White Center for Pediatric Infectious Diseases and Global Health, Indiana University School of Medicine, Indianapolis, IN USA; 2 Department of Pediatrics, Indiana University School of Medicine, Indianapolis, IN USA; 3 Jaramogi Oginga Odinga University of Science and Technology, Bondo, Kenya; 4 Academic Model Providing Access to Healthcare (AMPATH), Eldoret, Kenya; 5 Division of Infectious Diseases, Department of Medicine, Indiana University School of Medicine, Indianapolis, IN USA

**Keywords:** Asymptomatic malaria, Rapid diagnostic tests, HRP2

## Abstract

**Background:**

Accurate detection of asymptomatic malaria parasitaemia in children living in high transmission areas is important for malaria control and reduction programmes that employ screen-and-treat surveillance strategies. Relative to microscopy and conventional rapid diagnostic tests (RDTs), ultrasensitive RDTs (us-RDTs) have demonstrated reduced limits of detection with increased sensitivity to detect parasitaemia in symptomatic individuals. In this study, the performance of the NxTek^™^ Eliminate Malaria P.f test was compared with traditional microscopy and quantitative polymerase chain reaction (qPCR) testing methods of detection for *P. falciparum* parasitaemia among asymptomatic children aged 7–14 years living in an area of high malaria transmission intensity in western Kenya.

**Methods:**

In October 2020, 240 healthy children without any reported malaria symptoms were screened for the presence of *P. falciparum* parasitaemia; 120 children were randomly selected to participate in a follow-up visit at 6–10 weeks. Malaria parasitaemia was assessed by blood-smear microscopy, us-RDT, and qPCR of a conserved *var* gene sequence from genomic DNA extracted from dried blood spots. Sensitivity, specificity, and predictive values were calculated for field diagnostic methods using qPCR as the gold standard. Comparison of detectable parasite density distributions and area under the curve were also calculated to determine the effectiveness of the us-RDT in detecting asymptomatic infections with low parasite densities.

**Results:**

The us-RDT detected significantly more asymptomatic *P. falciparum* infections than microscopy (42.5% vs. 32.2%, P = 0.002). The positive predictive value was higher for microscopy (92.2%) than for us-RDT (82.4%). However, false negative rates were high for microscopy and us-RDT, with negative predictive values of 53.7% and 54.6%, respectively. While us-RDT detected significantly more infections than microscopy overall, the density distribution of detectable infections did not differ (P = 0.21), and qPCR detected significantly more low-density infections than both field methods (P < 0.001, for both comparisons).

**Conclusions:**

Us-RDT is more sensitive than microscopy for detecting asymptomatic malaria parasitaemia in children. Though the detectable parasite density distributions by us-RDT in our specific study did not significantly differ from microscopy, the additional sensitivity of the us-RDT resulted in more identified asymptomatic infections in this important group of the population and makes the use of the us-RDT advisable compared to other currently available malaria field detection methods.

## Background

Low-density *Plasmodium falciparum* infections serve as a reservoir for malaria and contribute to continued transmission of disease in areas of high and low prevalence [1]. However, field-based identification of asymptomatic *Plasmodium* parasitaemia (AP) remains a challenge, as many subclinical infections have parasite densities below the detection threshold of conventional field diagnostic tools [2, 3]. When assessed by quantitative nucleic acid amplification tests (NAATs), individuals with AP have a broad range of parasite densities, from less than one to several thousand parasites per microlitre of whole blood (pRBC/µL) [1, 3–6]. Depending on the specific assay and volume of blood used, quantitative NAATs can be highly sensitive with detection limits as low as 0.02 pRBC/μL [6, 7] and can identify mosquito-transmissible infections [4, 8]. However, due to the need for specialized equipment, high cost per sample, and required technical expertise, NAATs are primarily used in research settings and have only limited utility for routine malaria diagnosis in the field. Despite these limitations, quantitative NAATs can provide standardized benchmarks for determining the accuracy of field-based diagnostics.

In malaria-endemic areas of sub-Saharan Africa, resource-limited, rural areas typically have higher *P. falciparum* prevalence relative to geographically matched urban centres [9], highlighting the need for low-cost, field-deployable diagnostic tools. Blood smear microscopy is still the most widely used field diagnostic method in malaria-endemic settings due to its low supply cost, quantitative readout, and potential use for species-level parasite identification. However, blood smear microscopy remains resource-intensive in terms of time and trained personnel and is therefore limited mainly to clinical settings. The sensitivity of microscopy is also highly dependent on the training and experience of the microscopist, with relatively wide limits of reliable detection ranging from 50 to 500 pRBC/µL [10]. For active surveillance of AP, more scalable diagnostics are needed for practical implementation outside the clinic.

Conventional rapid diagnostic tests (RDTs) are rapid, highly portable, and easy to use, making them ideal for assessment of AP. There are more than 200 different conventional RDTs currently in use with a range of reliable detection of 15–100 pRBC/µL [10]. Screen-and-treat strategies aimed at reducing the burden of asymptomatic infections in multiple transmission settings have shown that conventional RDTs fail to detect a substantial portion of AP, presumably due to their limited sensitivity, which is only a modest improvement over microscopy [11–13]. Most notably, a community-based study in western Kenya used a World Health Organization (WHO)-recommended conventional RDT with a reported sensitivity of > 95% for samples with > 200 pRBC/µL [14] to identify positive malaria cases for subsequent treatment. Despite achieving high coverage in this study, the conventional RDT screen-and-treat strategy still missed up to 20% of infections that were detectable by quantitative real-time PCR [13].

For continued progress in malaria control and reduction, it will be critical to develop and evaluate new field-based tools capable of detecting low-density parasitaemia. The ultrasensitive Alere^™^/Abbot Malaria Ag P.F RDT, now called NxTek^™^ Eliminate Malaria Ag P.f (us-RDT), detects the *P. falciparum* histidine-rich protein 2 (HRP2) antigen and has an assessed limit of detection of 3 pRBC/µL in laboratory-grown cultures [15]. This us-RDT also detected more than half of infections with a qPCR parasite density of 0.1–1.0 pRBC/µL in human blood samples from areas of high and low malaria transmission [16]. A recent meta-analysis of cross-sectional field surveys that evaluated NxTek^™^ us-RDT in asymptomatic individuals estimates its sensitivity to be 56.1%, an increase from 44.3% sensitivity of conventional RDTs used on the same participant samples [17]. While the current field-based sensitivity of the us-RDT leaves room for improvement, the lower limit of detection and improved performance over conventional RDTs makes the NxTek^™^ us-RDT a potentially valuable tool for the detection and treatment of AP in both high and low transmission areas.

The primary aim in this study was to evaluate the diagnostic accuracy of the us-RDT for field detection of AP in a school-aged pediatric population. Previous community surveys for AP using us-RDT included adults, who may have lower parasite densities due to the development of immunity to blood-stage infection. It was hypothesized that amongst the paediatric study participants, who are likely to have higher parasite densities [18], the us-RDT would demonstrate higher sensitivity and specificity for detecting AP than in a general population, would approach quantitative NAAT detection of conserved multigene regions in *P. falciparum* [3, 18], and outperform field microscopy for infections with 1–100 pRBC/µL. To test these hypotheses, the performance of the NxTek^™^ us-RDT was compared with (1) blood smear microscopy and (2) qPCR of DNA extracted from dried blood spots to identify AP in an area of high malaria transmission in western Kenya. Over 98% of malaria infections contain *P. falciparum* parasites in this endemic area [19], which make this more sensitive HRP2-based test a potentially valuable tool for detection. Additionally, a subset of qPCR-positive samples were evaluated for *hrp2* gene deletion mutations, to assess the accuracy of the HRP2-based NxTek^™^ us-RDT in sub-Saharan Africa where the spread of *hrp2* deletion mutations has recently been reported [20, 21]. This study provides additional field validation of a promising diagnostic tool in a paediatric demographic group that is an important target for ongoing malaria reduction efforts [22, 23].

## Methods

### Study population and recruitment

The study was conducted from October 2020 to December 2020 at the Gobei Health Centre and a local school in the Ajigo sublocation, Bondo sub-county, Siaya County, western Kenya. From a community-wide census, children aged 7–14 years were randomly recruited in an age-stratified manner to participate in a study to evaluate the effect of asymptomatic *P. falciparum* parasitaemia on cognition [24]. A total of 264 children were recruited at school and invited to enroll in the study by visiting the Gobei Health Centre. Consent and enrollment of the 240 participating children was completed at the health center. Children with signs or symptoms of malaria at the time of enrollment were excluded from this study. A total of 240 children were enrolled in the study, with 30 children per 1-year age strata. A random number generator was used to select 120 participants among the 240 enrolled for a follow-up visit at 6–10 weeks. Children were fluent in either Dhuluo or English. Primary caregivers provided informed consent, and children 13 years and older also provided assent for participation.

### Ethical review

Ethical review and approval for this study was given by the Institutional Review Board at Indiana University, the Institutional Research Ethics Committee at Moi Teaching and Referral Hospital in Eldoret, Kenya. A research permit was obtained by the Kenya National Commission for Science, Technology and Innovation.

### Participant sampling

Peripheral whole blood samples were collected from the pediatric participants via venipuncture into an EDTA Vacutainer tube (Becton, Dickson, and Company, Franklin Lakes, NJ) using sterile procedures. Immediately after phlebotomy, the residual whole blood within the butterfly collection device, which does not contain anticoagulant, was applied as drops to the us-RDT and used to prepare thick and thin microscopy blood smears and dried blood spots (DBS) on Whatman 903 protein saver cards (Cytiva, Marlborough, MA). The filled Vacutainer tube was centrifuged to separate plasma from blood cells.

### Detection of asymptomatic malaria

Identification of AP in the field was assessed within 48 h of sample collection by light microscopy by counting the number of parasites per 200 leukocytes on a thick smear using two independent readings, with a third reading for slides with discordant results. Thin blood smears were used for *Plasmodium* species identification. Only microscopy-positive individuals were provided with full treatment doses of malaria medication based on guidelines of the Kenyan Ministry of Health. Participants that were us-RDT positive, but negative for malaria by microscopy were advised to visit their local clinic for re-evaluation because us-RDTs have not yet been approved for clinical use.

Ultrasensitive RDT testing was done onsite using the ultra-sensitive NxTek^™^ Eliminate Malaria Ag P. falciparum HRP2 antigen RDT (Abbott Rapid Diagnostics, South Africa [25]) according to the manufacturer’s instructions. Briefly, us-RDTs were stored at room temperature, which was within the 1–30 °C storage conditions provided by the manufacturer, and one drop of fresh whole blood was applied to the sample area of the RDT followed by four drops of diluent into the square diluent well. Tests were used within the specified expiration deadline (12 months) placed on a flat surface and read after 20 min of development.

Assessment of AP in the laboratory was done by extraction of DNA from DBS followed by qPCR. Total DNA was extracted from six 0.32-cm diameter circles punched from each DBS sample using a QIAmp 96 Blood Kit (Qiagen, Valencia, CA). Briefly, DBS were punched into deep tube 96-well plates. Tissue lysis (ATL) buffer was added, and samples were incubated at 85 °C for 10 min to disrupt red blood cell membranes. Samples were then incubated with proteinase K for 1 h at 56 °C to degrade red blood cell proteins. Samples were further incubated with AL buffer for 10 min at 70 °C and then added to the QIAmp Mini spin columns. After centrifugation, subsequent DNA isolation with ethanol and washing steps were followed per the Qiagen protocol. DNA was eluted from the 96-well column plate twice in 30 µL of EDTA buffer. Extracted DNA was quantitatively evaluated for *P. falciparum* parasitaemia in duplicate by qPCR targeting a conserved sequence region identified on members of the var gene family as previously described [3, 18]. Discordant results were re-run in triplicate and required two or more parasite positive results of the three replicate wells to be considered qPCR-positive for AP.

### Detection of hrp2 by PCR

DNA samples that contained more than 2.0 parasites/µL and were malaria-positive by qPCR, but negative by us-RDT were tested for the presence/absence of the *hrp2* gene using a previously published nested endpoint PCR method [26]. When possible, a similar number of samples that were positive by both us-RDT and NASBA for each parasitaemia range were also tested for presence/absence of *hrp2*. Of 80 total samples selected for amplification, there were 25 low- (< 10 parasites/µL), 35 medium- (10–100 parasites/µL), and 20 high-density (> 100 parasites/µL) infections. A starting volume of 2.0 µL of DNA was used as a template for round 1 of PCR to amplify *P. falciparum hrp2* exon 2. Laboratory malaria strains 3D7 and HB3 were used as positive controls since these strains are known to contain the full-length *hrp2* gene. Laboratory strain Dd2, which has a full-length *hrp2* deletion*,* was used as a negative control. Round 1 product was diluted 1:5 prior to round 2 of amplification. A total of 7.0 µL of final product was loaded on a 2.0% agarose gel and visualized using ethidium bromide and ultraviolet light. Negative samples were rerun with 4.0 µL of starting DNA template.

### Statistical analysis

Statistical analysis was done using R software version 4.0.5. Histograms of qPCR-determined parasite densities were plotted to assess the distribution of the data. Sensitivity, specificity, positive and negative predictive values for us-RDT and microscopy were evaluated using qPCR as the reference standard for true positives and true negatives. Area under the receiver operating characteristic curve (AUROC) plots were evaluated separately for all participants at enrollment, and for all randomly selected follow-up participants. The AUROC was also used to determine the sensitivity vs. specificity thresholds that would provide the best prediction of performance for each diagnostic method [27]. Nonparametric tests with a significance level of 0.05 were performed to test the null hypotheses that pRBC distributions were equal among test types (Kruskal–Wallis) with follow-up pairwise testing (paired Wilcoxon rank sum, with Benjamini–Hochberg correction). Two-sample tests of proportions were used to determine whether the proportion of positives from us-RDT was equivalent to that of microscopy and qPCR. Pearson’s correlation was used to assess qPCR parasite density compared with participant age.

## Results

### Performance of us-RDT in asymptomatic children in a high transmission area

**Fig. 1:**
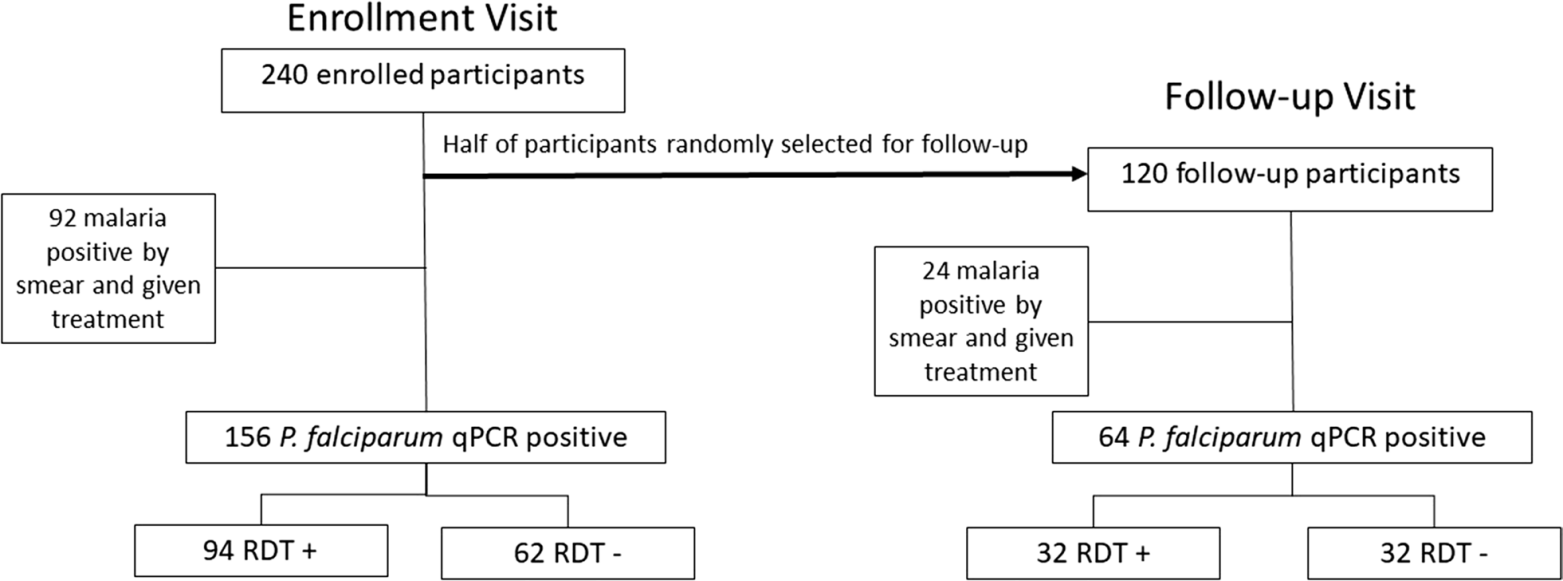
Study design. Pediatric study participants were recruited for this study from a local school in Ajigo in Siaya County, Kenya. A total of 240 participants were enrolled at the Gobei health Center, of which 120 were randomly selected for the follow-up group. Follow-up visits occurred 6–10 weeks after enrollment. The number of participants receiving treatment and number positive by test type are included in the diagram

To evaluate the performance of the NxTek^™^ us-RDT, 240 paediatric participants ages 7–14 years old who had no symptoms of malaria were tested for parasitaemia. Half of study participants (n = 120) were randomly selected for a follow-up visit 6–10 weeks after enrollment (Fig. 1). Across all 360 samples collected, the us-RDT detected AP infections in 153 (42.5%) compared to 116 (32.2%) detected by microscopy and 220 (61.1%) detected by qPCR (Table 1).

**Table 1:** Comparison among malaria testing methods

	Enrollment (n = 240)	Follow-up (n = 120)	All samples (n = 360)
qPCR positive, n (%)	156 (65.0%)	64 (53.3%)	220 (61.1%)
qPCR density median [IQR] (calculated pRBCs/μL)	19.5 [1.5, 281.1]	13.3 [1.6, 164.4]	17.1 [1.5, 248.9]
Microscopy positive, n (%)	92 (38.3%)	24 (20.0%)	116 (32.2%)
us-RDT positive, n (%)	108 (45%)	45 (37.5%)	153 (42.5%)
Micro sensitivity (%) [95%CI]	53.8 [50.3, 57.3]	35.9 [26.4, 45.4]	48.6 [46.9, 50.3]
us-RDT sensitivity (%) [95%CI]	60.3 [54.6, 66.0]	50.0 [ 50.0, 50.0]	57.3 [53.4, 61.2]
Micro specificity (%) [95%CI]	90.5 [79.1, 100]	98.2 [80.6, 100]	93.6 [84.0, 100]
us-RDT specificity (%) [95%CI]	83.3 [73.0, 93.6]	76.8 [63.7, 89.9]	80.7 [72.6, 88.8]
Micro PPV (%) [95%CI]	91.3 [79.8, 100]	95.8 [78.7, 100]	92.2 [82.7, 100]
us-RDT PPV (%) [95%CI]	87.0 [76.1, 97.9]	71.1 [ 59.6, 82.7]	82.4 [74.1, 90.1]
Micro NPV (%) [95%CI]	51.4 [49.3, 53.5]	57.3 [ 50.4, 64.1]	53.7 [50.9, 56.5]
us-RDT NPV (%)[ 95%CI]	53.0 [49.9, 56.1]	57.3 [50.4, 64.1]	54.6 [51.5, 57.7]

At enrollment, 92 of the 240 children (38.3%) had AP detectable by microscopy, compared to 108 (45.0%) positive cases by contemporaneous us-RDTs. Children with microscopy-detectable AP were given anti-malarial medication. Retrospective qPCR from dried blood spots detected AP in 156 participant samples (65.0%; Table 1). Ultrasensitive-RDT did not detect significantly more AP cases than microscopy (P = 0.069) and detected significantly fewer cases relative to qPCR (P < 0.001). Among qPCR-positive participants, the median parasite density at enrollment was 19.5 parasites/µL, IQR [1.5, 281.1], compared to 160.9 parasites/µL, IQR [19.5, 1022.9] in those detected by us-RDT, and 235.4 parasites/µL, IQR [31.9, 1108.3] detected by microscopy. Parasite density as determined by qPCR did not correlate with age (Spearman ρ = 0.092, P = 0.082).

After initial enrollment, half of study participants were randomly selected for follow-up at 6–10 weeks (Fig. 1). Among these 120 children, the 47 participants diagnosed with AP by microscopy were treated at enrollment. During the follow-up visits, 24 of 120 participants (20.0%) were diagnosed with AP by microscopy. Forty-five children (37.5%) tested positive by us-RDT, and 64 (53.3%) had detectable parasitaemia upon qPCR testing (Table 1). At the follow-up visit, us-RDT detected significantly more AP infections than microscopy (P = 0.0014) and significantly fewer than qPCR (P = 0.0069). Participants with AP at follow-up had a median parasite density of 13.3 parasites/µL, IQR [1.6, 164.4]. The difference between AP densities was not statistically significant between enrollment and follow-up visit (Wilcoxon test, P = 0.12).

Due to its low limit of detection, qPCR was used as the reference standard for determining whether an individual had a true *P. falciparum* infection. During both enrollment and follow-up visits the sensitivity of us-RDT was higher than microscopy (60.3% vs. 53.8% and 50.0% vs. 35.9%, respectively; Table 1). However, the specificity of us-RDT was consistently lower than microscopy (83.1% vs. 90.5% at enrollment, 76.8% vs. 98.2% at follow-up; Table 1). Thus, the us-RDT detects more true positives than microscopy, but also produces more false positives.

**Fig. 2:**
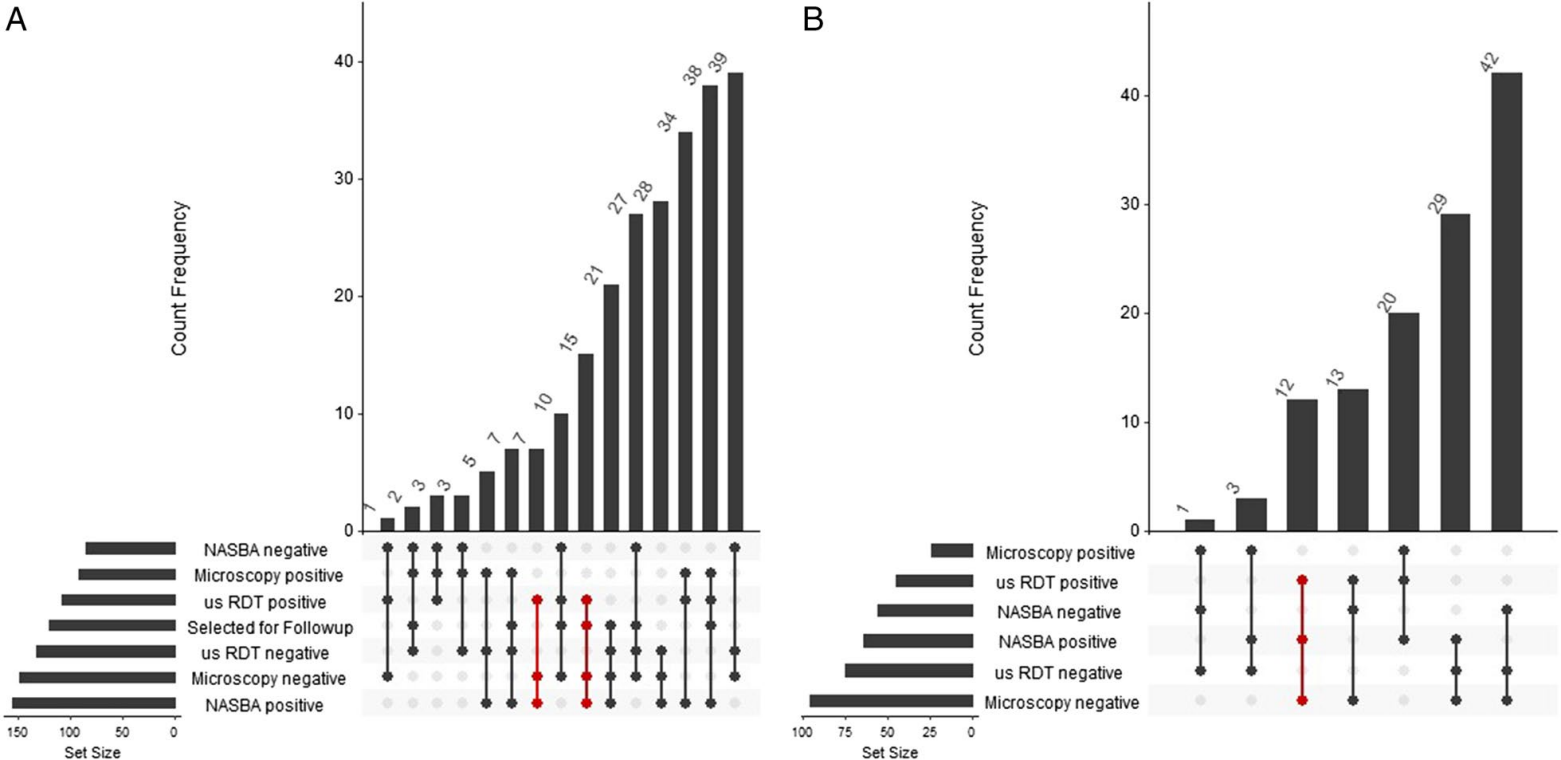
Intersection of results from three tests for *Plasmodium* parasitaemia in asymptomatic children. Upset plot of the overlap of participants testing positive by microscopy, us-RDT, and qPCR for enrollment (**A**) and follow-up at 6–10 weeks (**B**). The number of participants with each test result is presented as the set size. Connecting dots indicate the combination of test results, which are plotted by increasing count frequency. Red connections highlight qPCR positive samples detected by us-RDT but missed by microscopy

The positive predictive values (PPV) and negative predictive values (NPV) were comparable between microscopy and us-RDT at enrollment but differed at follow-up visits. Both microscopy and us-RDT had high PPVs and low NPVs at enrollment visit (Table 1). For follow-up visits, microscopy had notably higher PPV than us-RDT: 95.8% vs. 76.8%. Both field detection methods had the same NPV during follow-up visits (Table 1). Overall, there were many more detected cases of AP by qPCR than by either field detection method (Fig. 2). For both enrollment and follow-up visits, more than one quarter of participants with detectable AP by any method were detected only by qPCR (28.6% at enrollment, 37.2% at follow-up).

**Fig. 3:**
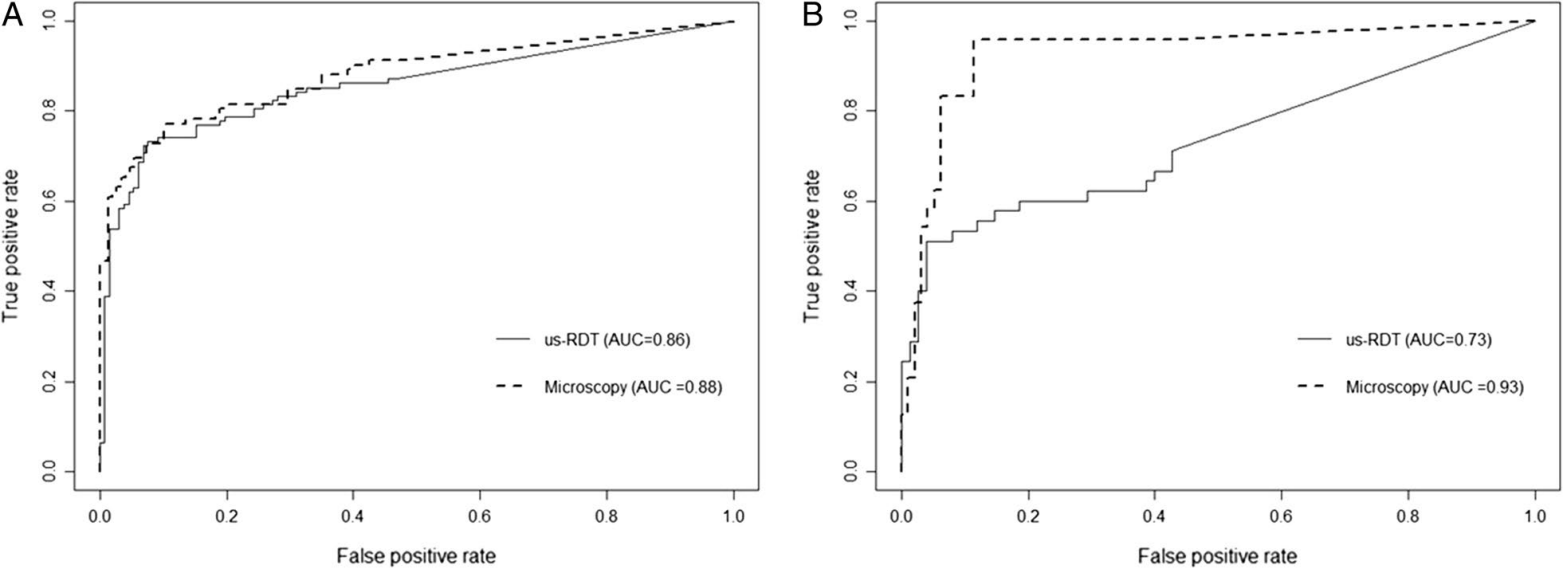
Performance of microscopy and us-RDT based on parasite density. Area under the receiver operator curves (AUROC) comparing microscopy and us-RDT for enrollment (**A**) and follow-up (**B**) visits. qPCR quantitative values were used to determine true positive and negatives

The values determined from qPCR were used as the reference standard for calculating the area under the receiver operator curve (AUROC) for all participant samples at enrollment and follow-up visits. The AUROC for us-RDT and microscopy were then compared to evaluate the performance of these two field-based tests (Fig. 3). At the enrollment visit, microscopy and us-RDT had similar performance [AUROC = 0.881, 95% CI (0.833, 0.929) vs 0.856, 95% CI (0.805, 0.907), P = 0.475; Fig. 3A]. At enrollment similar prediction thresholds were used for both field tests. For follow-up visits, the AUROC for microscopy indicated better performance than us-RDT [0.930, 95% CI (0.866, 0.993) vs. 0.731, 95% CI (0.634, 0.828), P = 0.001; Fig. 3B]. Based on the areas under the curve, and the overall shape, a sensitivity threshold for detection of true positives by microscopy of > 0.80 would be appropriate to prevent the inclusion of excess false positives during the prediction of test outcome. A lower true positive threshold of approximately 0.50 would be more appropriate to exclude excess false positives during predictions of us-RDT results from the follow-up visit.

**Fig. 4:**
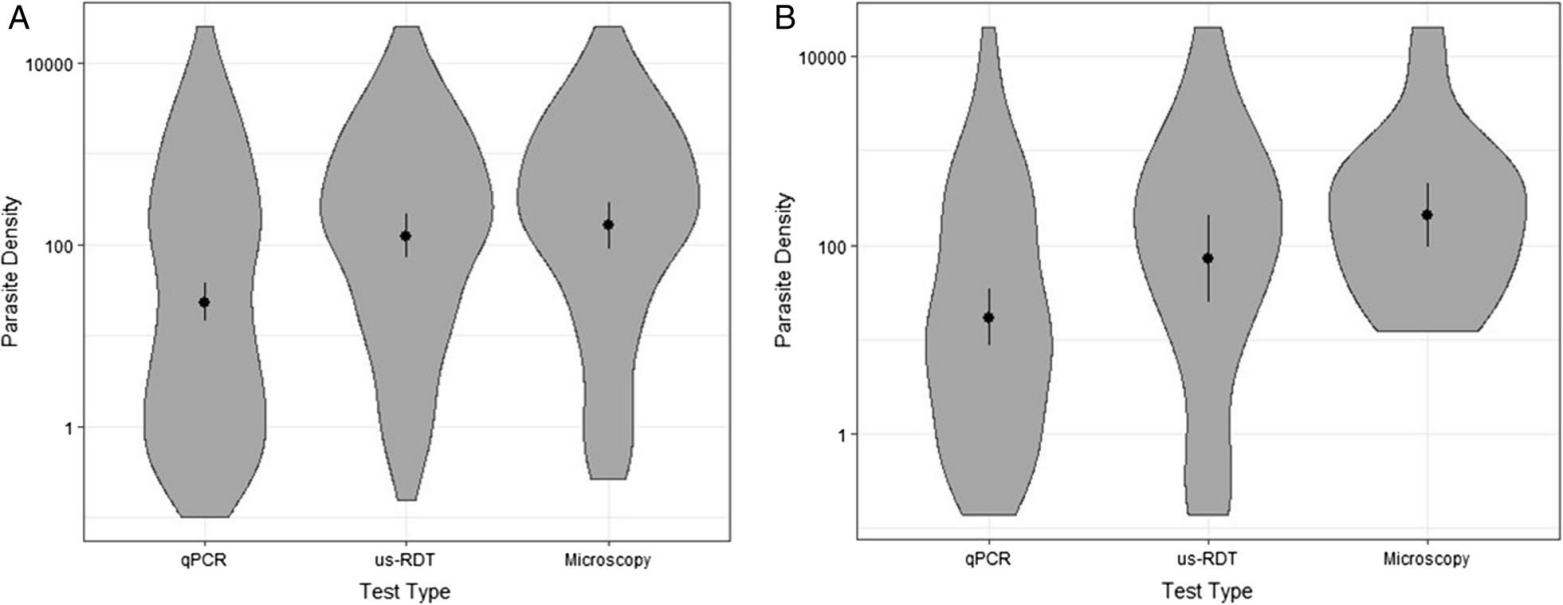
Detection of AP by parasite density. Violin plots of the parasite density, median and IQR of positive tests for each type of test at enrollment (**A**) and 6- to 10-week follow-up visit (**B**)

To compare the distribution of detectable parasite densities with each of the three methods, qPCR parasite densities for positive tests were assessed for both enrollment and follow-up samples (Fig. 4). At both enrollment and follow-up visits the density distributions were statistically different (Kruskal–Wallis P < 0.001). Specifically, qPCR-detected parasitaemia had lower values than microscopy (P < 0.001) and us-RDT (P < 0.001), but the distribution of detected parasitaemia was not statistically different between us-RDT and microscopy (P = 0.21). The parasite densities of infections detected at enrollment vs. follow-up visits did not differ indicating that each methodology worked similarly well at both visits, specifically the median [IQR] for infections detected at enrollment vs. follow-up by qPCR was 19.5, [1.5, 281.1] vs. 13.3, IQR [1.6, 164.4], P = 0.18, by us-RDT was 160.9 [19.5, 1022.9] vs. 150.8 [16.0, 708.5], P = 0.11, and by microscopy was 235.4 [31.9, 1108.3] vs. 237.5 [50.5, 718.2], P = 0.07 (Table 1, Fig. 4).

### Decreased us-RDT sensitivity is not due to hrp2 gene deletions

To determine whether performance of the us-RDT was affected by parasite gene deletions in histidine-rich protein 2 (*hrp2*), which is the primary antigen detected by this RDT, we amplified the *hrp2* genomic region of us-RDT–negative, qPCR-positive samples collected enrollment and follow-up. No gene product was detected in six samples (7.4%). Three of these *hrp2* negative samples were among the 31 us-RDT–negative, qPCR-positive samples tested (9.7%), whilst the remaining three *hrp2* negative were positive for parasitaemia by us-RDT. Five of the six *hrp2* negative had low-parasite densities of < 10 pRBC/μL, which may have been nearing the reliable limit of detection for the endpoint PCR used to target *hrp2*. Detection of *hrp2* in 80% (20/25) of low-density samples is greater than the sensitivity of us-RDT (8/25, 32%), indicating that lower sensitivity of us-RDT in these infections was not due to high abundance of *hrp2* deletions. Furthermore, 54/55 medium- and high-density infections had endpoint PCR-detectable *hrp2*. The results suggest that *hrp2* negative samples may have been near the limit of detection and may have been amplified using a more sensitive assay.

### Evaluation of ACT treated and untreated participants after 6–10 weeks

Forty-seven participants of the study who were randomly assigned to the follow-up group had microscopy-detected AP at enrollment and were given a treatment dose of anti-malarial medication based on Kenyan Ministry of Health guidelines. An additional 16 and 34 AP infections were identified by us-RDT and qPCR, respectively. However, since the us-RDT used in the study has not been approved for clinical use in Kenya, and qPCR was performed after the visit, participants with AP detected by these assays were not provided treatment at the time of their visit. Of those treated during their enrollment visits, 10/47 (21.3%) were positive by microscopy at the follow-up visit (Table 2). A total of 18/47 (38.3%) and 25/47 (53.2%) of participants given treatment were positive at follow-up by us-RDT and qPCR, respectively. As determined by two-proportion Z score tests, the positivity rate for previously treated participants was not statistically different from participants who did not receive treatment during the enrollment visit 14/73 (19.2%) by microscopy (P = 0.78), 27/73 (40.0%) by us-RDT (P = 0.89), and 39/73 (53.3%) by qPCR (P = 0.98)—indicating that treatment during enrollment did not impact infection status at the follow-up timepoint (Table 2). Parasite density at the follow-up visit was not significantly different between participants who were treated vs. untreated during enrollment (P = 0.37). Parasite density was also not different between enrollment and follow-up visits for the 45 participants who had qPCR detectable AP at both visits (pairwise Wilcoxon rank sum, P = 0.12). Comparing follow-up parasite densities between children who received and did not receive treatment at the enrollment visit was also not statistically significant (P = 0.15).

**Table 2:** Comparison of participants assigned to follow-up group

	At enrollment (n = 120)	At follow-up, not treated at enrollment (n = 73)	At follow-up, treated at enrollment (n = 47)
qPCR positive, n (%)	81 (67.5%)	39 (53.4%)	25 (53.2%)
qPCR density median [IQR] (calculated pRBCs/μL)	29.3 [1.3, 267.7]	11.7 [2.3, 164.4]	18.0 [1.14, 131.0]
Microscopy positive, n (%)	47 (39.2%)	14 (19.2%)	10 (21.3%)
us-RDT positive, n (%)	63 (52.5%)	27 (37.0%)	18 (38.3%)
Micro sensitivity (%) [95%CI]	55.6 [49.6, 61.6]	33.3 [20.0, 46.6]	40.0 [27.2, 52.8]
us-RDT sensitivity (%) [95%CI]	65.4 [55.5, 75.3]	48.7 [45.0, 52.4]	52.0 [46.3, 57.7]
Micro specificity (%) [95%CI]	94.9 [77.9, 100]	97.1 [74.8, 100]	100 [71.4, 100]
us-RDT specificity (%) [95%CI]	74.4 [61.9, 86.9]	76.5 [59.8, 93.2]	77.3 [56.2, 98.4]
Micro PPV (%) [95%CI]	95.7 [78.6, 100]	92.9 [71.7, 100]	100 [71.4, 100]
us-RDT PPV (%) [95%CI]	84.1 [69.3, 98.9]	70.4 [55.7, 85.1]	72.2 [53.1, 91.3]
Micro NPV (%) [95%CI]	50.7 [48.6, 52.8]	55.9 [48.0, 63.8]	59.5 [47.0, 72.0]
us-RDT NPV (%) [95%CI]	50.9 [48.5, 53.3]	56.5 [48.2, 64.8]	58.6 [46.7, 70.5]

## Discussion

The purpose of this study was to assess the use of us-RDTs for identification of AP among children in an area with high malaria transmission. Prior studies evaluating us-RDTs have examined its efficacy in the laboratory [6, 15], and as a tool for detection of AP in populations living in areas with relatively high or low malaria transmission [16, 17, 28]. This field-based assessment adds new knowledge about the effectiveness of this diagnostic tool, when used with blood samples among asymptomatic children ages 7–14 years old in an area with high malaria transmission. While prior studies have evaluated this us-RDT in screen and treat studies that have included young children and adults, in areas with high malaria transmission such as the one in our study, school-aged children older than 5 years of age are beginning to develop malarial immunity, and thus are more likely to be asymptomatic if infected. The children included in our study are of particular relevance for continued malaria reduction as recent age-based assessments of parasite burden have identified school-aged children, ages 5–14 years old, as primary contributors to transmission [23, 29, 30]. In this study, there were no differences in parasite density based on age, indicating that, in this high transmission area, many children have developed partial immunity to infection by the time they reach the age of seven, indicating that this entire age group is of importance for asymptomatic transmission. An increased ability to identify AP within this pediatric subpopulation could promote additional interventions to address the reservoir of silent but transmissible infections.

In the current study, the NxTek^™^ Eliminate Malaria Ag P.f us-RDT identified more AP infections than microscopy, but significantly fewer than qPCR at both enrollment and follow-up visits. When using qPCR testing as the reference for true positives and true negatives, both us-RDT and microscopy had higher sensitivity during enrollment. While the difference between parasite densities of participants with AP was not statistically significant between study time points, it is notable that both the median and third quartile for parasitaemia were higher at enrollment than during the follow-up visit. This subtle difference in parasite density very likely impacted the sensitivity and AUROC curves of the us-RDT at follow-up, as only 45 children selected for follow-up were positive for AP by us-RDT at both visits. Treatment of microscopy-positive children at enrollment did not have an impact on the actual number of children infected with AP during the follow-up visit. Similar proportions of children (~ 53%) in the treated and untreated groups were positive for AP by qPCR at follow-up. Lower parasite density distributions at follow-up likely resulted from a combination of factors. Microscopy-negative, qPCR-positive participants at enrollment did not receive treatment and may represent individuals who were already both controlling parasitaemia to sub-microscopic levels and tolerating their infection. Participants who were malaria-positive at enrollment and received treatment possibly acquired additional anti-parasitic immunity as a result of their initial infection. Due to the high transmission dynamics in the study area, where people are potentially exposed to multiple infectious mosquito bites per week, it is not surprising that over half of the children treated for malaria presented with AP during their follow-up visits 6–10 weeks after receiving curative treatment. These participants were likely reinfected between enrollment and follow-up. The lower parasitaemia in these individuals at follow-up may have been due to the additional immunity gained during prior infections. These lower-density infections during follow-up visit, though not statistically significant, very likely had an impact on the sensitivity of both us-RDT and microscopy detection of AP.

Overall sensitivity of us-RDT was better than microscopy, and this increase resulted in reduced specificity. However, negative results on either microscopy or us-RDT were not reliable at enrollment or follow-up visit. Participants with negative test results by either method were just as likely to be positive for AP, indicating that a potentially important part of the silent infectious reservoir would go undetected by available field tests. An increased understanding of whether very low-density AP infections contribute to onward transmission is still needed to determine whether detecting and treating such infections would lead to community-wide reductions in malaria prevalence.

In our assessment of *hrp2* gene deletions, we detected the gene in all but six samples. All samples with undetected *hrp2* had low or midrange densities (< 100 pRBC/µL). Notably, the HRP2-antigen based us-RDT was positive in half of the *hrp2* PCR-negative samples, which could be due to cross-reactivity with HRP3 antigen. Such cross-reactivity has been previously observed in conventional RDTs [31]. As the majority of *hrp2*-negative infections had fewer than 10 pRBC/µL, we also speculate that samples with undetected *hrp2* by endpoint PCR may be positive for *hrp2* if evaluated by a more sensitive laboratory detection method that can detect HRP2 antigen in very low density infections [32]. These results suggest that *hrp2* deletions are rare and do not contribute to effectiveness of the us-RDT in this region. This is consistent with the results of recent studies in Kenya which have found few *hrp2* deletions [20].

This study was limited by the number of study participants, especially those that were included in the follow-up evaluation. While the study was conducted in an area of high transmission with a total of 240 participant, both enrollment and follow-up visits were conducted in only half (n = 120) of the enrolled children. Of these, 45 children (21 treated and 24 not treated during enrollment) were positive for AP by qPCR at both timepoints. Based on these results, this study had ~ 75% power (β = 0.263) to detect differences in parasite densities between enrollment and follow-up. Confirmation of the current findings in a larger longitudinal cohort will be needed. While a recent meta-analysis suggests that sensitivity and specificity of the us-RDT is lower than we report for symptomatic adults in low- transmission areas [17], this difference in performance may be related to the lower level of malaria exposure and immunity in the meta-analysis group. The children in this study, who spanned 7–14 years in age, had similar parasite densities across all age strata, which may represent the acquisition of near-maximal immunity against blood-stage infections in a highly intense transmission setting. Another potential explanation for lower than expected specificity of the us-RDT would be delayed clearance of HRP2 antigen after parasite clearance [33] which could have resulted in false positive us-RDT test results compared to qPCR.

The NxTek^™^ Eliminate Malaria Ag P.f us-RDT is marketed as a field-based tool for areas aiming to “reduce the malaria reservoir in the community and thereby drive down transmission” [25]. This study demonstrates that this us-RDT detects AP at lower densities than microscopy but not may have adequate sensitivity to identify all mosquito-transmissible infections in this area. A cross-sectional study in an area of moderate malaria transmission in Papua New Guinea recently showed that infections with < 1 parasites/µL and detectable only by ultrasensitive qRT-PCR, as compared to standard PCR, rarely have high enough gametocyte density for transmission [34]. While the us-RDT was able to detect 82% of infections with parasite densities greater than 10 pRBC/µL, it only identified one-quarter of infections with less than 10 pRBC/µL. Despite this higher than anticipated detection limit, models that used data from many different transmission settings to simulate the reduction of transmission achieved in various active case detection scenarios suggest that us-RDT sensitivity may be appropriate for screen-and-treat strategies in low transmission areas nearing elimination if implemented at high coverage [35]. In areas with high transmission, model simulations indicate mass drug administration is still advisable compared to screen-and-treat studies [35]. In this study area where malaria transmission remains high, and many children have asymptomatic infections with densities < 10 parasites/µL, in which the us-RDT performed comparably to other high-incidence areas and model simulations [17]. While it did not approach the sensitivity of qPCR-based detection methods in this population, we would still recommend the use of the us-RDT compared to currently available field diagnostics due to the increase in number of infections identified compared to the conventional clinic-based microscopy diagnosis of malaria infection.

## Data Availability

The data generated and analyzed during this study are included in this published article and its supplementary information files where applicable. Additional study data are available from the corresponding author on reasonable request.
